# HTR1A a Novel Type 1 Diabetes Susceptibility Gene on Chromosome 5p13-q13

**DOI:** 10.1371/journal.pone.0035439

**Published:** 2012-05-01

**Authors:** Samina Asad, Pernilla Nikamo, Alexandra Gyllenberg, Hedvig Bennet, Ola Hansson, Nils Wierup, Diabetes Incidence in Sweden Study Group, Annelie Carlsson, Gun Forsander, Sten-Anders Ivarsson, Helena Larsson, Åke Lernmark, Bengt Lindblad, Johnny Ludvigsson, Claude Marcus, Kjersti S. Rønningen, Jan Nerup, Flemming Pociot, Holger Luthman, Malin Fex, Ingrid Kockum

**Affiliations:** 1 Neuroimmunology Unit, Department of Clinical Neurosciences, Karolinska Institutet, Stockholm, Sweden; 2 Department of Molecular Medicine and Surgery, Karolinska Institutet, Stockholm, Sweden; 3 Diabetes and Celiac Unit, Department of Clinical Sciences, Lund University, Malmö University Hospital, Malmö, Sweden; 4 Diabetes and Endocrinology, Department of Clinical Science, Lund, University, Malmö University Hospital, Malmö, Sweden; 5 Neuroendocrine cell biology, Department of Clinical Science, Lund, University, Malmö University Hospital, Malmö, Sweden; 6 Lund University Diabetes Center, Lund, Sweden; 7 Department of Pediatrics, Lund University Hospital, Lund, Sweden; 8 Department of Pediatrics, the Queen Silvia Children’s Hospital, Göteborg, Sweden; 9 Division of Pediatrics, Department of Clinical and Experimental Medicine, Diabetes Research Center, Linköping University Hospital, Linköping, Sweden; 10 Division of Pediatrics, Department of Clinical Science, Intervention and Technology, National Childhood Obesity Center, Karolinska Institutet, Stockholm, Sweden; 11 Department of Pediatric Research, Oslo University Hospital, Rikshospitalet, Oslo, Norway; 12 Steno Diabetes Center, Gentofte, Denmark; 13 Glostrup Research Institute, University Hospital Glostrup, Glostrup, Denmark; University of Bremen, Germany

## Abstract

**Background:**

We have previously performed a genome-wide linkage study in Scandinavian Type 1 diabetes (T1D) families. In the Swedish families, we detected suggestive linkage (LOD≤2.2) to the chromosome 5p13-q13 region. The aim of our study was to investigate the linked region in search for possible T1D susceptibility genes.

**Methodology/Principal Findings:**

Microsatellites were genotyped in the Scandinavian families to fine-map the previously linked region. Further, SNPs were genotyped in Swedish and Danish families as well as Swedish sporadic cases. In the Swedish families we detected genome-wide significant linkage to the *5-hydroxytryptamine receptor 1A (HTR1A*) gene (LOD 3.98, p<9.8×10^−6^). Markers tagging two separate genes; the *ring finger protein 180* (*RNF180*) and *HTR1A* showed association to T1D in the Swedish and Danish families (p<0.002, p<0.001 respectively). The association was not confirmed in sporadic cases. Conditional analysis indicates that the primary association was to *HTR1A.* Quantitative PCR show that transcripts of both *HTR1A* and *RNF180* are present in human islets of Langerhans. Moreover, immunohistochemical analysis confirmed the presence of the 5-*HTR1A* protein in isolated human islets of Langerhans as well as in sections of human pancreas.

**Conclusions:**

We have identified and confirmed the association of both *HTR1A* and *RFN180,* two genes in high linkage disequilibrium (LD) to T1D in two separate family materials. As both *HTR1A* and *RFN180* were expressed at the mRNA level and *HTR1A* as protein in human islets of Langerhans, we suggest that *HTR1A* may affect T1D susceptibility by modulating the initial autoimmune attack or either islet regeneration, insulin release, or both.

## Introduction

The incidence of Type 1 diabetes (T1D) is rapidly increasing around the world [1] and Sweden has one of the highest incidence rates of T1D [2]. T1D is an autoimmune disease which is characterised by gradual destruction of insulin producing beta-cells located in the pancreas.

The detailed aetiology of T1D is still unknown, yet it is understood that both environmental and genetic factors contribute to disease susceptibility. Studies have identified more than 40 susceptibility loci for T1D [3]. The *Major Histocompatibility Complex (MHC)* on chromosome 6 is known to be the key T1D susceptibility region accounting for more than 50% of the total genetic risk. Other T1D susceptibility genes include the *insulin* gene (chromosome 11p5), the *CTLA-4* gene (chromosome 2q33), *IL2RA* (chromosome 10p15), *IFIH1* (chromosome 2q24) and the *PTPN22* gene (chromosome 1p13) [3]. However, the identified T1D susceptibility genes and gene regions do not explain all of the genetic risk. This could be due to gene-gene and gene-environment interactions, epigenetic effects but also due to so far unidentified susceptibility genes.

We have in a previous genome-wide linkage study detected suggestive linkage (LOD<2.2) to the chromosome 5p13-q13 region in a Scandinavian T1D family material [4]. In the present study we narrowed the region of linkage and identified associated markers in the *5-hydroxytryptamine (serotonin) receptor 1A (HTR1A)* and *the ring finger protein 180* (*RNF180)* genes. We then confirmed this association in independent materials. Further we have detected *HTR1A* and *RNF180* expression in human pancreatic islets of Langerhans.

## Methods

### Ethics Statement

The ethics committees at Karolinska Insitutet, Umeå University, Copenhagen University and Oslo University have approved blood sampling for the purpose of genetic analyses for the current study. Oral or written consent was obtained from the patients, controls or their guardians.

Further, the ethics committee at Lund University has approved the sampling of pancreases of organ donors who have approved the use of their organs for medical research according to the organ donor register.

### Patients and Controls

#### Scandinavian families

The Swedish families consist of 184 multiplex and 9 simplex Swedish families, including a total of 200 affected T1D sib-pairs. The Danish family material consist of 147 multiplex and 5 simplex families with 175 affected sib-pairs, while the Norwegian material consists of 77 multiplex and 2 simplex families with 89 affected sib-pairs [4] (Table 1).

**Table 1:** T1D data materials.

Category	Swedish	Danish	Norwegian
Sib pairs:			
Affected	200	175	89
Unaffected	53	135	23
Discordant	277	287	123
Multiplex families	184	147	77
Simplex	9	5	2
Age at onset median(range)	14 (0–53)	13 (0–71)	12 (1–52)
	**DISS2**	**BDD**	**MS**
Cases	778	2300	–
Controls	836	–	527
T1D at onset (%)	76.2	95	–
Age/age onset median	25	10	
(range)	(15–34)	(0–19)	(18–70)

* Only healthy controls were used from the Epidemiological Investigations in Multiple Sclerosis (EIMS) study.

#### Diabetes Incidence Study in Sweden 2 (DISS2)

The Diabetes Incidence Study in Sweden consists of 778 patients with diabetes and 836 matched controls identified through the diabetes incidence study register. The incident DISS2 patients were between 15 and 34 years. At follow-up visits 528 patients were classified with T1D [5]. The remaining 250 patients were classified as type 2 diabetes patients, secondary diabetes or unclassified diabetes patients and were therefore excluded from our study.

#### Better Diabetes Diagnosis Study (BDD)

The BDD cohort consists of 2742 newly diagnosed diabetes patients. All patients were less than 18 years of age at the time of diagnosis. Patients were recruited between May 2005 and September 2009 from 40 pediatric clinics in Sweden [6]. At follow up, 95% of the patients were classified as T1D while the remaining 5% were classified with type 2, MODY, secondary, “other” or “unknown” type of diabetes. All patients of non-European descent were excluded from the cohort. In the current study we have included 2300 T1D patients from the BDD cohort.

#### Multiple Sclerosis (MS) controls

We have included 527 healthy controls between the ages of 18–70 years from an ongoing population based case control study of multiple sclerosis (MS) called EIMS (Epidemiological Investigations in Multiple Sclerosis) in our analysis. Controls were matched for age, gender and residential area to incident MS cases throughout Sweden [7].

### Genetic Fine-mapping of Chromosome 5

#### Microsatellite genotyping

Fine mapping of chromosome 5 was performed by typing microsatellites in the Scandinavian families. A total of 40 microsatellite markers were selected from NCBI (http://www.ncbi.nlm.nih.gov/), GDB (http://gdbwww.gdb.org/), and Marshfield (http://research.marshfieldclinic.org/genetics/). PCR conditions and quality controls were as described [4].

#### SNP genotyping

For genotyping of Single Nucleotide Polymorphisms (SNPs) a total of 67 SNPs were selected with an average spacing of 10–30 kb and a minor allele frequency above 0.3. A lower rare allele frequency was only accepted for SNPs within genes. Fifteen SNPs were chosen in possible functional candidate T1D susceptibility genes in the 5p13-q13 region (Table S1). All SNPs were genotyped either using Pyrosequencing (Pyrosequence Inc., Sweden) [8] or the TaqMan method (Applied Biosystems Inc., Sweden) as described [9].

All SNPs in the MS controls were genotyped using the Human660-Quad chip as described [10].

#### DNA sequencing

Due to the lack of identified SNPs in *HTR1A*, sequencing of the gene and its flanking regions was performed using the ABI Prism 3730 Genetic Analyser method (Applied Biosystems Inc., Sweden). DNA from six T1D patients (four patients carrying a *HTR1A* T1D risk haplotype and two carrying a non-risk haplotype) and five controls (two controls with the T1D risk haplotype and three without) were sequenced. Haplotypes were defined through previously typed SNPs using the GeneHunter program. The 11 756 bp long DNA sequence was divided into approx. 1 000 bp segments. Primer sequences are available on request. Primers were obtained from DNA Technology A/S (Risskov, Denmark). Sequencing was performed in ABI the Prism 3730 Genetic Analyser and the results were analysed in the SeqScape software, v. 2.5 (Applied Biosystems Inc., Sweden).

### Statistical Analysis

All microsatellite markers were mapped to the chromosome 5p13-q13 region using CRIMAP [11]. Mendelian consistencies, excess of homozygosity (Hardy-Weinberg equilibrium) and allele frequencies for the analysed SNPs and microsatellites in the families were estimated using the zGenStat program (H. Zazzi, unpublished). Linkage was assessed by non-parametric linkage methods (ALLEGRO [12]). Single- and multi-point linkage analyses were performed using the exponential model with equal weighing. Combined analysis of linkage and association as well as identifying SNPs responsible for linkage was performed in the Linkage and Association Modeling in Pedigrees (LAMP) software program [13]. The LD structure and tagging of SNPs were analyzed in Haploview [14]. LD blocks were identified using all three block definitions with similar results. Hardy-Weinberg equilibrium was tested among controls only. Single SNP and haplotype association as well as conditional association analysis were assessed using the Unphased computer program (version 3.0.6) [15]. Study cohort was used as a confounder in the combined association analyses.

### Imputation

Imputation of the rs6295, rs356570 and rs6880454 SNPs among the MS controls was performed using the MaCH v. 1.0.16 analysis program with default settings [16]. Previous SNP genotyping results from the MS Genome wide association (GWAS) study were used. Quality controls of typed SNPs included checking for call-rates (per sample and per SNP), heterozygosity, recent shared ancestry, non-European ancestry, MAF and deviation from Hardy-Weinberg equilibrium [10].

### Human Islet Donor Material

Human islets were obtained from the Nordic Network for Clinical Transplantation, Uppsala and distributed by the Human tissue laboratory at Lund University Diabetes Center, Malmö, Sweden.

### Quantitative-PCR of HTR1A and RNF180 mRNA from Human Islets of Langerhans

mRNA was prepared from human islets of Langerhans from a total of 10 healthy age and gender matched donors using mRNA Easy Plus mini kit, (Qiagen, Hilden, Germany). cDNA was obtained by reverse transcription (Maxima ™ First Strand cDNA Synthesis Kit, Fermentas, Thermo Scientific, Sweden). The mRNA levels were quantified using a Probe/Rox Real-Time PCR (Maxima™ Probe/ROX qPCR Master Mix (2X), Fermentas, Thermo Scientific, Sweden) with an ABI PRISM 7900 (Applied Biosystems, Inc., Sweden), and assays-on-demand were employed for *HTR1A* (Hs 00265014) and *RNF180* (Hs 00400379) (Applied Biosystems Inc., Sweden). Each sample was run in duplicate, and the transcript quantity was normalized to the mRNA level of *cyclophilin A* (Hs 01565700, *PPIA*), *polymerase 2 (*Hs 00172187, *POL2A) and hypoxanthine guanine phosphoribosyl transferase (*Hs 01003267, *HPRT)* (Applied Biosystems).

### Tissue Preparation and Immunohistochemistry

For histochemical analyses human islets and biopsies from human pancreas were fixed overnight in Stefanini’s solution (2% paraformaldehyde and 0.2% picric acid in 0.1 M phosphate buffered saline, pH 7.2), rinsed thoroughly in Tyrode solution containing 10% sucrose and frozen on dry ice. Sections (10 µm) were cut and thaw-mounted on slides. Antibodies were diluted in phosphate buffered saline (pH 7.2) containing 0.25% bovine serum albumin and 0.25% Triton X-100. Sections were incubated with primary antibodies (goat anti- 5HTR1A; code sc-1459; dilution 1∶100 (Santa Cruz Biotech. Inc., CA USA), guinea pig anti-proinsulin; code 9003; dilution 1∶2560; EuroDiagnostica, Malmö, Sweden), and rabbit anti-glucagon; code 7811; dilution 1∶5000 (EuroDiagnostica) overnight at 4°C in moisturizing chambers. The specificity of immunostaining was tested using primary antisera pre-absorbed with homologous antigen (100 µg of peptide per ml antiserum at working dilution). Immunofluorescence was examined in an epi-fluorescence microscope (Olympus, B×60). By changing filters the location of the different secondary antibodies in double staining was determined. Images were captured with a digital camera (Nikon DS-2Mv).

### Computational Analysis

The web based software RAVEN (www.cisreg.ca/cgi-bin/RAVEN/a) was used for detecting potential transcription factor binding sites.

## Results

### Fine-mapping of Chromosome 5p13-q13 and HTR1A Sequencing

The region on chromosome 5 (5p13-q13) which previously showed suggestive linkage [4] was narrowed down by genotyping additional microsatellites and SNPs located in the area, in separate cohorts.

**Figure 1:**
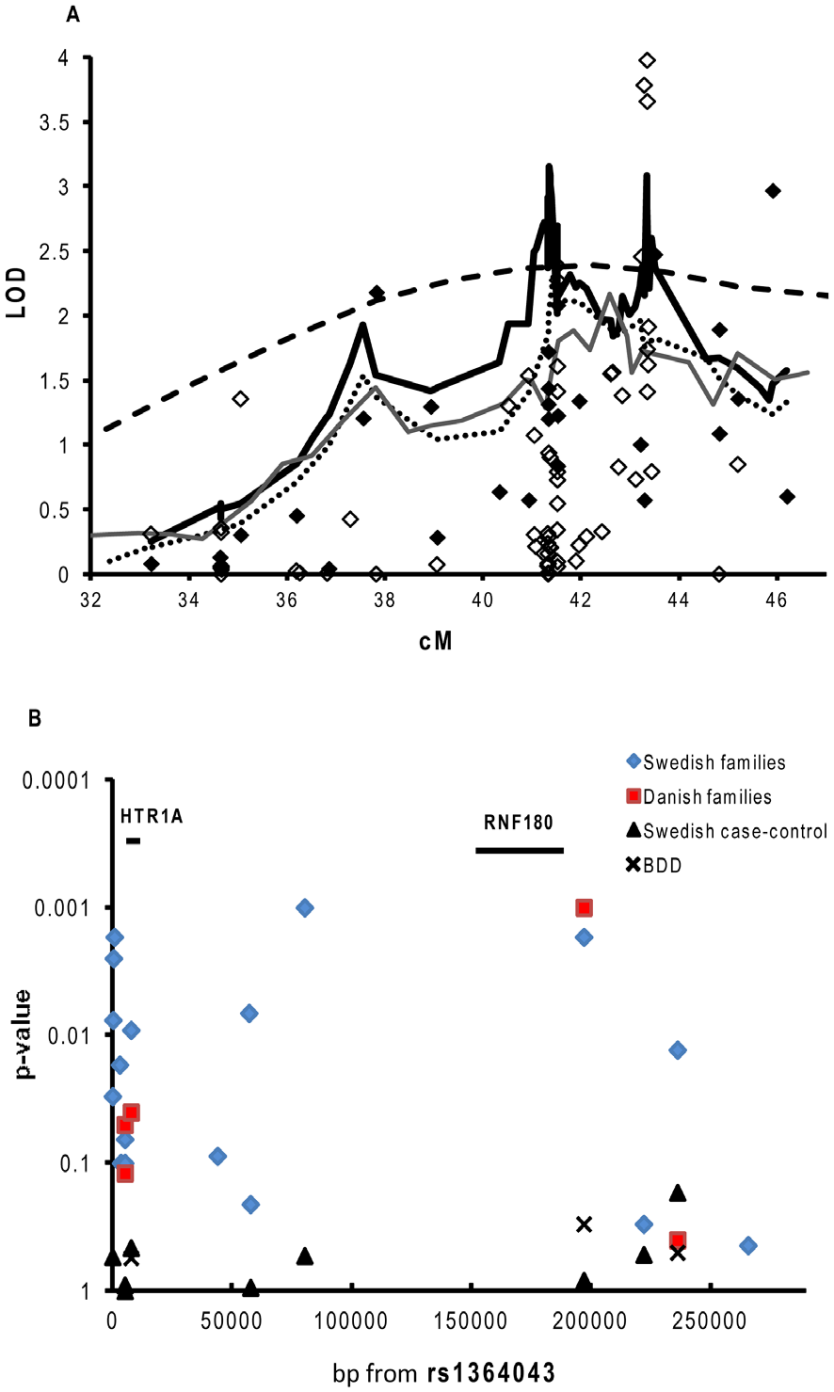
a–b. Linkage and association analysis in Scandinavian families and case-control cohort. Linkage analysis of T1D on chromsome 5 in Swedish, -Danish and -Norwegian multiplex families (a). The dashed line represents multipoint linkage from the original scan in the Swedish, Danish and Norwegian families [4]. The thin dark grey line represents the “fine mapping” including 36 microsatellites in all Scandinavian families. A region between D5S407 and D5S428 (at approx 41.5 cM in figure) showed a linkage of LOD 2.16. The dotted line represents the Swedish families using all 40 microsattelites (multipoint). Here, D5S2000 showed strongest linkage (LOD 2.70). The black diamonds represent the “fine mapping” single point analysis in the Swedish families. In the singlepoint analysis for the Swedish families, D5S2048 showed strong linkage (LOD 2.97). The black thick line represents linkage in Swedish families to T1D on chromosome using an extra 4 microsattelites and 61 SNPs, reveiling three linked peaks where the most strongly linked region was the 5p13-q13 (at approx 43 cM on figure) region (LOD 2.7 for rs6295). In the singlepoint analysis using all 61 SNPs and 4 extra microsattelites (white diamonds) rs878567 and rs6295 showed genome-wide significant linkage (LOD 3.9 and LOD 3.65 respectively). Linkage was calculated using the Exponential Equal Weighting model in the Allegro program.SNP association for the Swedish sporadic cases and controls and Swedish and Danish multiplex families (b) was calculated from rs1158292 (63 001 317 bp) to rs6880454 (63 5028 02 bp). Swedish families (diamonds). Danish families (squares), DISS2 (triangles) and BDD (crosses). Association analysis was carried out in the Unphased program. For the BDD cohort, association was calculated using controls included in the Swedish (MS) EIMS study.

We first typed an additional 36 microsatellites in the Scandinavian families. A region of 20.6 cM between the microsatellites D5S407 and D5S428 showed strongest support for linkage (LOD 2.16, p<0.0008). In the single point analysis, using the exponential equal weighting model, D5S2048 reached a LOD of 2.97 (p<0.0002). The added evidence for linkage to this region provided by the Danish and Norwegian families was modest (LOD 0.68 p<0.04 and LOD 0.22 p<0.16). We therefore decided to perform further analysis in the Swedish families by typing four additional microsatellites. D5S2000 showed strongest linkage in a multipoint analyses (LOD 2.70, approx 41.5 cM, Figure 1a).

We next chose to genotype 61 evenly distributed SNPs across the 50 695 kb–63 532 kb region which was the region showing strongest evidence of linkage. Genotyping results revealed three linked regions (Figure 1a). The region showing strongest linkage (5p13-q13) contained the *HTR1A* gene. Two SNPs; rs878567 and rs6295, which are located on either side of *HTR1A,* displayed genome wide significant linkage of LOD 3.98, p<9.8×10^−6^ and LOD 3.66, p<2×10^−5^ (Figure 1a). Also, rs749099 located downstream of *HTR1A* showed significant linkage (LOD 3.78, p<1.5×10^−5^).

In the Swedish families, significant association was observed for the rs6295 SNP (p<0.01) which is located in very close proximity to *HTR1A* (1019 bp upstream of *HTR1A*). Also, several other SNPs in the *HTR1A* region showed association to T1D (Table 2 and Figure 1b), suggesting that *HTR1A* is a T1D susceptibility gene. Additionally, it was seen that rs356570, 190 kb upstream of *HTR1A* is associated to T1D (p<0.002).

**Table 2:** Association to the chromosome 5p13 region in Swedish multiplex/simplex families.

Marker name	Location bp	Minor Allele	Minor allele frequency	Call rate %	p-value
rs1158292	63 001 317	*A*	0.38	93	0.2
rs1364043	63 266 735	*G*	0.22	89	0.03
rs970453	63 266 983	*C*	0.46	87	0.008
rs72767932	63 267 175	*G*	0.14	88	0.003
rs1423691	63 267 546	*C*	0.48	84	0.002
rs749099	63 269 720	*T*	0.45	94	0.02
rs749098	63 270 176	*G*	0.20	85	0.1
rs878567	63 271 875	*C*	0.50	91	0.06
rs6449693	63 271 902	*A*	0.50	95	0.1
rs6295	63 274 449	*G*	0.49	90	0.01
rs1364041	63 324 375	*G*	0.12	97	0.2
rs382098	63 310 581	*C*	0.48	89	0.08
rs749100	63 323 746	*C*	0.41	95	0.007
rs356562	63 346 993	*A*	0.4	95	0.001
rs356570	63 463 673	*G*	0.37	89	0.002
rs12697015	63 4886 95	*C*	0.15	98	0.3
rs6880454	63 502 802	*T*	0.08	95	0.01
rs11949052	63 532 300	*A*	0.14	98	0.4

SNP location (bp) was obtained from “HapMap Data Rel 19/phasell Oct 05, on NCBI B34 assembly, dbSNP b124” in HapMap.

Minor allele frequencies and p-values for the Swedish multiple/simplex families were obtained from Unphased v. 3.0.6. Hardy-Weinberg (H–W) values were calculated amongst controls.

When the LAMP software was used to confirm our observed linkage for rs6295, suggestive linkage of LOD 2.4 (p<0.01) was observed for the rs6295 SNP. Further, when test for association was performed for rs6295 a LOD of 3.1 and p<1.6×10^−4^ was observed, suggesting that this SNP is responsible for the observed linkage.

**Figure 2:**
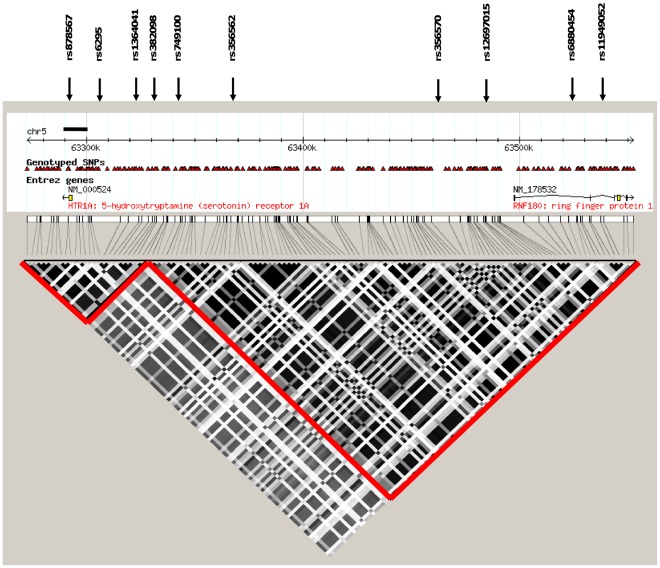
LD plot for HTR1A and RNF180. LD-block of the *HTR1A* and *RNF180* regions was obtained from Haploview using HapMap data. The linkage disequilbrium measure shown is r^2^ and the block definition is solid spine defined by Gabriel *et al.,* [35]. *HTR1A* and *RNF180* are situated in two separate blocks (r^2^ = 0.91 between the two blocks). When LD for the two associated SNPs (rs356570 and rs6880454) is calculated we observe a D́ value of 1 while r^2^ is 0.021. The SNPs in bold were typed to verify the involvement of *RNF180* to T1D susceptibility. Rs12697015 tags three other SNPs located in the *RNF180* downstream region. rs6880454 tags 17 additional SNPs on either side of *RNF180*. rs11949052 only tags for itself. Standard settings were used for tagging anlysis in Haplowiev. The bold lines indicate the *HTR1A* sequenced area.

No SNPs in the coding sequence of *HTR1A* were found in Ensemble or dbSNP. Therefore, in the search for possible unidentified SNPs, sequencing of the *HTR1A* gene and promoter was carried out. In an 11 756 bp long region around *HTR1A*, we sequenced 9 low risk haplotypes and 10 high risk haplotypes identified by haplotype association in the Swedish famililes. Through the sequencing, six previously identified and genotyped SNPs (rs1364043, rs1423691, rs749099, rs878567, rs6449693 and rs6295) were confirmed. Further, three SNPs which had not been published in a public data base at the time of the study, were identified (rs1364043, rs970453 and rs749098) and genotyped in the Swedish families. Using the Haploview program for LD block identification, it was seen that all of these SNPs are located within the same LD block (Figure 2). No SNPs in the coding sequence of the *HTR1A* gene were identified.

Using the Haploview program and genotypes in the 63 280 kb to 63 560 kb region from individuals with European ancestry from the HapMap project, we saw that rs356570 tags for 71 SNPs located further upstream of *HTR1A.* A number of these SNPs are located within or around the *RNF180* gene, suggesting that this gene could be involved in T1D susceptibility as well. It was also seen that the region between *HTR1A* and *RNF180* has very high LD (Figure 2).

To test the involvement of *RNF180* in the susceptibility to T1D, another three SNPs in or around *RNF180* were genotyped in the Swedish family material (Figure 1b). Genotyping analysis revealed significant association between T1D and rs6880454 (p<0.01 Table 2).

### Confirmation in Independent Cases and Controls

For confirmation of association, we genotypes markers in, Danish families and two collections of sporadic Swedish T1D cases; DISS2 and BDD material (Table 3).

**Table 3:** Association to the chromosome 5p13 region in Danish multiplex/simplex families, BDD cohort and the Swedish case-control material.

Marker name	Danish families			Swedish-Danish families	Swedish case-control				BDD cohort		MS controls		Pooling of data
					Patients	Controls			Patients		Controls		
					n = 778	n = 836			n = 2700		n = 527		
	Minor allele frequency	Call rate %	p-value	p-value	Genotypefrequency% *AA/AB/BB*	Genotypefrequency% *AA/AB/BB*	Call rate%	p-value	Genotypefrequency% *AA/AB/BB*	Call rate%	Genotypefrequency% *AA/AB/BB*	p-value	p-value
rs1364043					59.5/35.3/5.1	61.4/33.6/4.9	93	0.5					
rs878567	0.50	95	0.05	0.015	25.6/49.9/24.5	24.4/51.8/23.8	89	0.9					
rs6449693	0.50	93	0.06	0.04	25.6/50.2/24.1	25.9/49.6/24.4	81	0.9					
rs6295	0.50	92	0.04	0.003	27.5/50.9/21.6	26.6/49.6/23.8	89	0.4	27.2/50.0/22.9	95	26.6/50.1/23.4	0.4	0.01
rs1364041					77.7/20.6/1.7	78.2/19.7/2.0	83	0.8					
rs356562					37.5/49.3/13.3	37.3/47.2/15.5	94	0.5					
rs356570	0.38	95	0.001	8.5×10^−5^	38.8/48.6/12.6	39.5/46.5/14.0	94	0.8	40.8/45.3/13.9	97	41.5/44.4/14.1	0.6	0.003
rs12697015					73.1/25.3/1.6	71.9/26.0/2.2	93	0.5					
rs6880454	0.07	91	0.5	0.02	82.8/15.5/1.7	83.5/15.1/1.4	70	0.2	82.3/16.8/1.1	95	83.1/16.1/0.8	0.5	0.05

Allele frequencies and p-values for the Danish multiplex-simplex families and p-values for the Swedish cases-control and BDD studies were obtained from Unphased v. 3.0.6. P-values for the BDD cohort were obtained using controls from the Swedish MS case-control study.

Genotype frequencies and p-values for the genotype test were obtained by using chi2 test.

In the Danish families, rs6295 showed modest association (p<0.04). However, a stronger association was observed between T1D and rs356570 (p<0.001). No individual SNP showed association to T1D (Table 3, Figure 1b) in the Swedish DISS2 or BDD materials. Pooling of all four materials revealed that both rs6295 and rs356570 were associated with T1D (p<0.01 and p<0.003 respectively). Further, rs6880454 showed suggestive association of p<0.05.

Two haplotypes in the *HTR1A* and *RNF180* area in the Swedish and Danish families; a haplotype containing rs6295 (*C*) – rs356570 (*A*) – rs6880454 (*G*) showed positive association to T1D (p<0.002 and p<0.05 respectively) while haplotype rs878567 (*T*) – rs6449693 (*G*) – rs6295 (*G*) – rs356570 (*G*) showed protective association (p<0.03 and p<0.02, Table 4). No haplotype association was observed in the sporadic cases nor when all four materials were pooled.

**Table 4:** Haplotype association; Swedish – and Danish families.

Family material	Haplotype	p-value
**rs6295 -rs356570-**	**rs6880454**	
Swedish families	*C-A-G*	0.0013
Danish families	*C-A-G*	0.048
**rs878567-rs6449693-rs6295-**	**rs356570**	
Swedish families	*T-G-G-G*	0.025
Danish families	*T-G-G-G*	0.018

To see whether the two genes, *HTR1A* and *RNF180* were associated independently of each other, a conditional analysis was performed. When the two family materials were pooled, the rs6295 marker which maps close to *HTR1A* was associated independently of markers in *RNF180* (p<0.04). *RNF180* showed no association independently of *HTR1A*.

For *HTR1A* an analysis in the RAVEN program revealed that the rs6295 SNP positioned next to the *HTR1A* gene is located in a binding site for two transcription factors; Irf-1 and NRF-2.

### Quantitative-PCR of HTR1A and RNF180 mRNA from Human Islets of Langerhans

**Figure 3:**
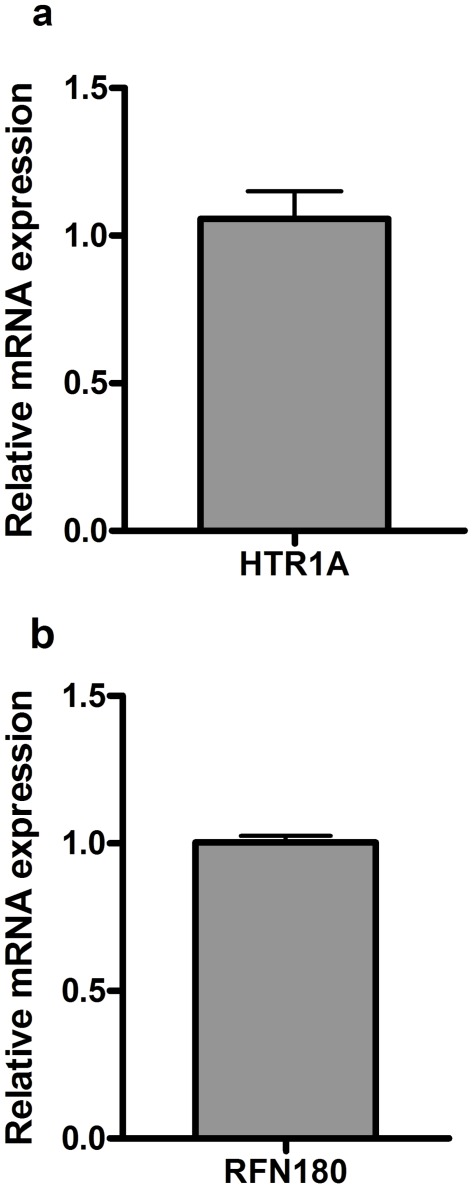
a-b. Q-PCR expression. Q-PCR expression for HTR1A (a) and RNF180 (b) from mRNA isolated from a total of 10 human islet donors. Data is presented as means of expression relative to the housekeeping genes *cyclophilin A* (*PPIA*), *polymerase 2 (POL2A) and hypoxanthine guanine phosphoribosyl transferase (HPRT)* +/− SEM.

mRNA was isolated and converted to cDNA from a total of 10 healthy islet donors. Relative mRNA expression of *HTR1A* (n = 6) (Figure 3a) indicates expression of *HTR1A* in human islets of Langerhans. Relative expression of *RFN180* (n = 4) was also detected in human islet donors (Figure 3b).

### HTR1A Protein in Human Beta Cells and Alpha Cells

**Figure 4:**
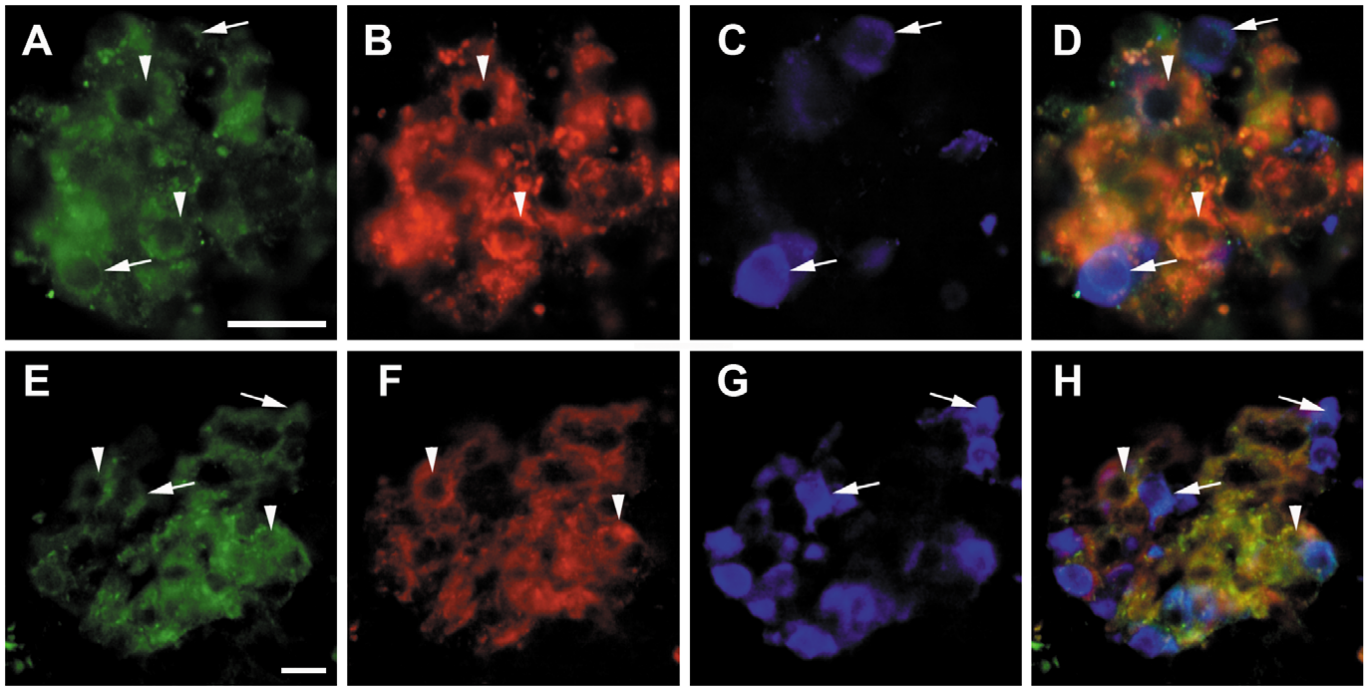
Human isolated islets and human pancreas triple immunostained for HTR1A, insulin and glucagon. Human isolated islets (A–D) and human pancreas (E–H) triple immunostained for HTR1A (A, E), insulin (B, F), and glucagon (C,G); merged in D and H. Arrow heads indicate beta cells with HTR1A immunoreactivity, arrows indicate alpha cells with HTR1A immunoreactivity. Scale bar = 20 um in A for upper panel, in E for lower panel.

**Figure 5:**
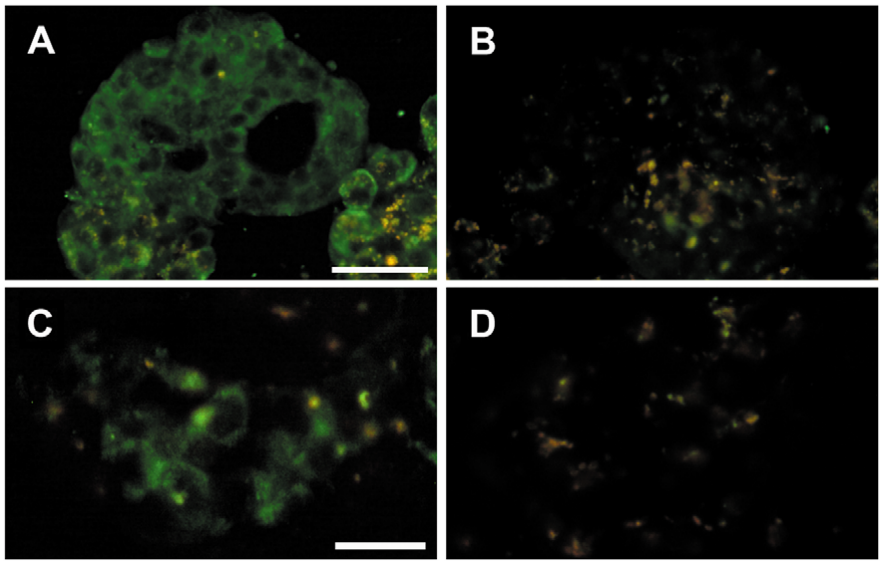
Human isolated islets and human pancreas with HTR1A antibodies preabsorbed with blocking peptide against HTR1A. Human isolated islets (A–B) and human pancreas (C–D). A and C immunostained for HTR1A. B and D the same islets as in A and C in consecutive sections immunstained with HTR1A antibodies preabsorbed with blocking peptide against HTR1A, demonstrating complete lack of immunoreaction. Scale bar = 50 um in A for upper panel, in 25 um in C for lower panel.

Staining of human beta and alpha-cells was performed to confirm results obtained from the q-PCR. Sections of human islets and human pancreatic specimens were triple immunostained for *HTR1A*, insulin and glucagon. This revealed that the *HTR1A* receptor is mainly expressed in beta-cells and alpha-cells (Figure 4). Importantly preabsorption with blocking peptide against *HTR1A* blocked all staining in both isolated human islets and in islets of human pancreatic sections (Figure 5).

## Discussion

We have in a previous genome-wide linkage study observed suggestive linkage of T1D to the 5p13-q13 region [4].

In previous reports several other autoimmune diseases such as Crohn’s disease [17], Autoimmune Thyroid disease (AITD) [18], Multiple Sclerosis (MS) [19], and Systemic lupus erythematosus (SLE) [20], have all been linked to the chromosome 5 region. Moreover, investigations indicate that *IL7R* which maps to the chromosome 5p13 region is associated to T1D as well as MS [21], [22].

The rs356570 SNP which is significantly associated to T1D in our Swedish and Danish families tags for several SNPs in the *HTR1A* and post *RNF180* region. Also, two haplotypes showed protective and positive association respectively in our families. No single SNP or haplotype association was detected in the Swedish sporadic cohorts. An explanation for this could be that there may be heterogeneity between familial and sporadic cases due to an enrichment of rare susceptibility genes with strong effect in the family materials. In the Swedish sporadic materials common susceptibility genes with low risk are likely to be more common. Further, the difference in association may also be due to changes in the environment. The multiplex families were collected a long time ago and it may be that changes in susceptible environmental factors have resulted in a change of importance of T1D susceptibility genes as has been suggested by us previously [6].

*HTR1A* and *RNF180* both show similar association to T1D in the multiplex families. However, in a conditional analysis, the *HTR1A* gene showed a slightly stronger association suggesting that *HTR1A* is the main T1D gene in this region. It should also be mentioned that since there is no LD between *IL7R* and the genes we have studied, our association cannot be due to *IL7Ŕ*s association to T1D (r^2^<0.01 and D́<0.02 between rs6897932 in *IL7R* and rs356570 in HapMap data). Further, GWAS studies performed by the Type 1 Diabetes Genetic Consortium (T1DGC) [3] and Welcome Trust Case Control Consortium (WTCCC) [23] do not detect any association of our studied region in their materials. The genotyped markers in the WTCCC study included our typed markers rs878567, rs382098 and rs356562 but not our most associated marker rs356570 [23]. All typed markers are in strong LD with each other (r^2^<0.91). In the GWAS study performed by WTCCC, sporadic rather than familial cases were used. In our study we only detect significant association in family materials. The use of sporadic versus familial cases may explain the contradictory results in our and the WTCCC study. Additionally, no SNPs which show association in our study were genotyped in the T1DGC study. It would therefore be interesting to genotype our studied SNPs in the multiplex T1DGC families to test if the lack of confirming the association of *HTR1A* in sporadic cases is due to a true heterogeneity between sporadic and multiplex cases.

We observe association for the rs6295 SNP in the multiplex families. Bioinformatic analysis indicate that rs6295 is located in a binding site for at least two transcription factors; Irf-1 which plays a role in regulating apoptosis and tumor suppression and NRF-2 which regulates cellular oxidative stress. It is therefore of interest to test the effect of rs6295 on expression of *HTR1A* in order to understand whether or not it is involved in the destruction of pancreatic beta-cells.

There are seven different types of serotonin receptors [24]. It has recently been reported that the *HTR2A* receptor is associated with Rheumatoid Arthritis [25].

*HTR1A* is a relatively short intronless gene consisting of only 1 268 bp. The gene is known to encode for a G-protein coupled receptor specific for serotonin which mediates cellular signaling via the amine serotonin (5-HT) [26]. The *HTR1A* receptor is expressed in many tissues but is mainly known to mediate signal transduction in neurons in the central nervous system [27] where it is involved in various complex behaviors such as appetite, sleep and aggression. Changes in serotonin signaling are associated with depression and suicide [28]. Expression of *HTR1A* has also been reported throughout the enteric nervous system and in the endocrine pancreas [29].

In the present study, we show that the 5-*HTR1A* protein is present in human pancreatic beta and alpha-cells. In accordance, we also show that *HTR1A* mRNA is expressed in human pancreatic islets. This provides an important functional correlation to the genetic data.

The neurotransmitter serotonin has been shown to be produced in pancreatic islets of several different species [30] and *in vitro* studies of rodent islet studies show that serotonin inhibits insulin secretion [31]. Moreover, systemic activation of *HTR1A* with sumatriptan in humans has a clear inhibitory effect on insulin secretion [32]. Whether this is due to a regulation at a central level or via a direct effect on the beta-cells, or both, remains to be elucidated. Furthermore, during pancreas regeneration, the expression of *HTR1A* is decreased while the insulin release is increased [33]. From this, it may be speculated that different *HTR1A* genotypes affects insulin release and could thereby be involved in the development of T1D. *HTR1A* has also been reported to play a role in the immune system. It has been reported that activated T-cells express high amounts of *HTR1A* while resting T-cells have low *HTR1A* expression [34]. Further, *HTR1A* is known to down regulate adenylate cyclase which in turn regulates T-cell functions such as production of IL-2 and cytotoxic T-cell activity [34]. Hence polymorphisms in the *HTR1A* gene may also affect the T-cell activity and thereby influencing the risk of developing T1D.

As mentioned earlier, two haplotypes containing rs356570 which maps upstream of both *HTR1A* and *RNF180* were identified in this study. It is therefore possible that a polymorphism in this region effects the expression of both *HTR1A* and *RNF180*. Since we in this study observe that *HTR1A* might be involved in T1D susceptibility independently of *RNF180*, we suggest that expression studies of *HTR1A* should be carried out in order to fully understand its function in T1D development. Also, further SNP genotyping in large case-control studies and sequencing should be carried out to confirm the association and determine which SNP in and around *HTR1A* is the main T1D associated marker.

## Supporting Information

Table S1 Typed microsatellites in the original genome-scan [4] and fine mapping. Typed microsatellites in the original scan have been run on all Scandinavian families, Norwegian, Danish and Swedish. Fine mapping has been performed on the Swedish and Danish families. All SNPs have been genotyped in the Swedish families. SNPs marked with * have been typed in the Swedish case control material DISS2, Δ have been typed in the Danish families and # indicates SNPs typed in the BDD material.
(DOC)
