# Supplementary material for: HTR1A a Novel Type 1 Diabetes Susceptibility Gene on Chromosome 5p13-q13
Source: PLoS One. 2012 May 1;7(5):e35439. doi: 10.1371/journal.pone.0035439 (PMC3341376; doi:10.1371/journal.pone.0035439)
Supplement: Table S1 — Typed microsatellites in the original genome-scan [4] and fine mapping. Typed microsatellites in the original scan have been run on all Scandinavian families, Norwegian, Danish and Swedish. Fine mapping has been performed on the Swedish and Danish families. All SNPs have been genotyped in the Swedish families. SNPs marked with * have been typed in the Swedish case control material DISS2, Δ have been typed in the Danish families and # indicates SNPs typed in the BDD material. (DOC) [file pone.0035439.s001.doc]

**Supplementary table.** *Table of all microsatellites run in*

*the original genome scan as well as in our paper*

| Marker name |  | Position | Gene region |
| --- | --- | --- | --- |
| D5S432 | Original Genome-scan | 10,745,924 |  |
| D5S416 | Original Genome-scan | 16,772,733 |  |
| D5S268 | Original Genome-scan | 17,472,929 |  |
| D5S419 | Original Genome-scan | 26,703,967 |  |
| D5S674 | Fine mapping | 33,636,455 |  |
| D5S426 | Original Genome-scan | 34,808,297 |  |
| D5S2022 | Fine mapping | 39,963,477 |  |
| D5S1457 | Fine mapping | 41,079,160 |  |
| D5S430 | Fine mapping | 41,417,746 |  |
| D5S822 | Fine mapping | 50,527,016 |  |
| rs3917084 | SNP genotyping | 50,695,344 | *ISL1* |
| rs991216 | SNP genotyping | 50,704,418 | *ISL1* |
| rs991217 | SNP genotyping | 50,704,686 | *ISL1* |
| rs1017 | SNP genotyping | 50,705,980 | *ISL1* |
| D5S1969 | Fine mapping | 53,258,717 |  |
| D5S2076 | Fine mapping | 54,222,747 |  |
| rs1396500 | SNP genotyping | 54,339,840 | *TRYP2* |
| rs1874469 | SNP genotyping | 54,788,077 | *PPAP2A1* |
| rs1373998 | SNP genotyping | 55,271,450 | *IL6ST* |
| D5S645 | Fine mapping | 55,321,620 |  |
| rs32510 | SNP genotyping | 55,584,206 |  |
| D5S633 | Fine mapping | 55,737,594 |  |
| rs1974851 | SNP genotyping | 55,997,112 |  |
| D5S407 | Original Genome-scan | 56,010,628 |  |
| D5S491 | Fine mapping | 56,416,940 |  |
| rs1552920 | SNP genotyping | 56,762,894 |  |
| D5S2102 | Fine mapping | 56,825,248 |  |
| D5S398 | Fine mapping | 57,584,872 |  |
| rs697134 | SNP genotyping | 57,770,017 |  |
| D5S2107 | Fine mapping | 58,165,690 |  |
| rs1553114 | SNP genotyping | 58,302,510 | *PDE4D1* |
| rs4415048 | SNP genotyping | 58,335,032 | *PDE4D1* |
| rs1023814 | SNP genotyping | 58,507,091 |  |
| rs153966 | SNP genotyping | 58,528,099 | *PDE4D1* |
| D5S2000 | Fine mapping | 58,630,820 |  |
| rs27170 | SNP genotyping | 58,634,391 |  |
| D5S2091 | Fine mapping | 58,638,644 |  |
| rs154023 | SNP genotyping | 58,652,447 |  |
| rs40126 | SNP genotyping | 58,666,818 |  |
| rs35285 | SNP genotyping | 58,672,441 |  |
| rs669240 | SNP genotyping | 58,705,991 |  |
| D5S2500 | Fine mapping | 58,712,926 |  |
| rs1379297 | SNP genotyping | 58,733,338 |  |
| rs992726 | SNP genotyping | 58,773,705 |  |
| rs2174624 | SNP genotyping | 58,784,032 |  |
| rs1156028 | SNP genotyping | 58,797,655 |  |
| D5S431 | Fine mapping | 58,816,512 |  |
| rs1605275 | SNP genotyping | 58,831,248 |  |
| rs167161 | SNP genotyping | 58,849,077 |  |
| rs953302 | SNP genotyping | 58,864,892 |  |
| rs159196 | SNP genotyping | 58,874,959 |  |
| rs294494 | SNP genotyping | 58,897,555 |  |
| rs746477 | SNP genotyping | 59,039,755 |  |
| D5S2080 | Fine mapping | 59,044,381 |  |
| rs877744 | SNP genotyping | 59,078,355 |  |
| D5S2018 | Fine mapping | 59,183,876 |  |
| D5S2071 | Fine mapping Swedish families | 59,226,473 |  |
| rs1533019 | SNP genotyping | 59,323,698 |  |
| rs719702 | SNP genotyping | 59,509,647 |  |
| rs37684 | SNP genotyping | 59,749,250 |  |
| rs27564 | SNP genotyping | 59,863,519 |  |
| D5S2945/2186 | Fine mapping Swedish families | *60,049,033* |  |
| rs1563907 | SNP genotyping | 60,072,885 |  |
| rs158930 | SNP genotyping | 60,241,408 |  |
| D5S624 | Fine mapping | 60,465,652 |  |
| rs1445979 | SNP genotyping | 60,760,224 |  |
| rs173780 | SNP genotyping | 60,919,086 |  |
| D5S1990 | Fine mapping | 60,990,937 |  |
| rs1020386 | SNP genotyping | 61,228,473 |  |
| rs27090 | SNP genotyping | 61,714,548 |  |
| rs1494725 | SNP genotyping | 61,997,816 |  |
| rs923963 | SNP genotyping | 62,055,527 |  |
| D5S76 | Fine mapping Swedish families | 62,234,967 |  |
| rs2221988 | SNP genotyping | 62,353,053 |  |
| rs424672 | SNP genotyping | 62,592,848 |  |
| rs585879 | SNP genotyping | 62,779,056 |  |
| D5S427 | Fine mapping | 62,918,978 |  |
| rs1158292 | SNP genotyping | 63,001,317 |  |
| rs1827540 | SNP genotyping | 63,016,933 |  |
| rs2059176 | SNP genotyping | 63,035,749 |  |
| rs1503529  D5S1956 | SNP genotyping  Fine mapping | 63,082,617  63,135,147 |  |
| rs1364043* | SNP genotyping | 63,266,735 |  |
| rs970453 | SNP genotyping | 63,266,983 |  |
| rs72767932 | SNP genotyping | 63,267,175 |  |
| rs1423691 | SNP genotyping | 63,267,546 |  |
| rs749099 | SNP genotyping | 63,269,720 |  |
| rs749098 | SNP genotyping | 63,270,176 |  |
| rs878567*∆ | SNP genotyping | 63,271,875 |  |
| rs6449693*∆ | SNP genotyping | 63,271,901 |  |
| rs6295*∆# | SNP genotyping | 63,274,449 |  |
| rs2032899 | SNP genotyping | 63,294,592 |  |
| rs382098 | SNP genotyping | 63,310,581 |  |
| rs749100 | SNP genotyping | 63,323,746 |  |
| rs1364041* | SNP genotyping | 63,324,375 |  |
| rs356562* | SNP genotyping | 63,346,993 |  |
| rs356570*∆# | SNP genotyping | 63,463,673 |  |
| rs12697015* | SNP genotyping | 63,488,695 |  |
| rs6880454*∆# | SNP genotyping | 63,502,802 |  |
| rs11949052 | SNP genotyping | 63,532,300 |  |
| D5S1359 | Fine mapping | 63,587,973 |  |
| D5S507 | Fine mapping Swedish families | 65,503,514 |  |
| D5S2089 | Fine mapping | 65,959,589 |  |
| D5S2072 | Fine mapping | 66,058,663 |  |
| D5S647 | Original Genome-scan | 66,263,034 |  |
| rs1697143 | SNP genotyping | 66,496,953 |  |
| rs1835019 | SNP genotyping | 66,599,713 |  |
| D5S2048 | Fine mapping | 66,642,764 |  |
| D5S2036 | Fine mapping | 66,970,522 |  |
| D5S2046 | Fine mapping | 67,254,575 |  |
| D5S629 | Fine mapping | 68,311,260 |  |
| D5S1988 | Fine mapping | 73,539,416 |  |
| D5S424 | Fine mapping | 76,241,792 | *CD180* |
| D5S1977 | Fine mapping | 76,398,576 | *CD180* |
| D5S2029 | Fine mapping | 81,541,344 |  |
| D5S428 | Original Genome-scan | 85,494,697 |  |
| D5S644 | Fine mapping | 95,886,767 |  |
| D5S409 | Original Genome-scan | 102,806,071 |  |
| D5S2501 | Fine mapping | 110,112,390 |  |
| D5S421 | Original Genome-scan | 112,939,359 |  |
| D5S210 | Original Genome-scan | 144,496,769 |  |
| D5S410 | Original Genome-scan | 152,803,485 |  |

***Supplementary table*.** Microsatellites in the original genome-scan [4] have been run on all Scandinavian families; Norwegian, Danish and Swedish. Fine mapping has been performed on the Swedish and Danish families. All SNPs have been genotyped in the Swedish families. SNPs marked with * have been typed in the Swedish case control material DISS2, ∆ indicates typed SNPs in the Danish families and # indicates SNPs typed in the BDD material.
